# Segmental chromosomal aberrations as the poor prognostic factor in children over 18 months with stage 3 neuroblastoma without *MYCN* amplification

**DOI:** 10.3389/fonc.2023.1134772

**Published:** 2023-02-14

**Authors:** Aleksandra Wieczorek, Katarzyna Szewczyk, Tomasz Klekawka, Joanna Stefanowicz, Marek Ussowicz, Grazyna Drabik, Katarzyna Pawinska-Wasikowska, Walentyna Balwierz

**Affiliations:** ^1^Department of Pediatric Oncology and Hematology, Medical College, Jagiellonian University, Krakow, Poland; ^2^Department of Pediatric Oncology and Hematology, University Children’s Hospital of Krakow, Krakow, Poland; ^3^Department of Medical Genetics, Institute of Pediatrics, Medical College, Jagiellonian University, Krakow, Poland; ^4^Department of Pediatrics, Pediatric Hematology and Oncology, Medical University of Gdansk, Gdansk, Poland; ^5^Department of Pediatric Bone Marrow Transplantation, Oncology, and Hematology, Wroclaw Medical University, Wroclaw, Poland; ^6^Department of Pathology, University Children’s Hospital of Krakow, Krakow, Poland

**Keywords:** stage 3, *MYCN* non-amplified neuroblastoma, segmental chromosomal aberrations, prognostic factor, neuroblastoma

## Abstract

**Introduction:**

Patients with stage 3 neuroblastoma (NBL) according to International Neuroblastoma Staging System (INSS) without MYCN amplification represent a heterogenous group with respect to disease presentation and prognosis.

**Methods:**

Retrospective analysis of 40 stage 3 patients with NBL without MYCN amplification was performed. The prognostic value of age at diagnosis (under 18 vs over 18 months), International Neuroblastoma Pathology Classification (INPC) diagnostic category and presence of segmental or numerical chromosomes aberrations were evaluated, as well as biochemical markers. Array comparative genomic hybridization (aCGH) for analyzing copy number variations and Sanger sequencing for ALK point mutations were done.

**Results:**

In 12 patients (two patients under 18 months), segmental chromosomal aberrations (SCA) were found and numerical chromosomal aberrations (NCA) were found in 16 patients (14 patients under 18 months). In children over 18 months SCA were more common (p=0.0001). Unfavorable pathology was significantly correlated with SCA genomic profile (p=0.04) and age over 18 months (p=0.008). No therapy failures occurred in children with NCA profile over or under 18 months or in children under 18 months, irrespective of pathology and CGH results. Three treatment failures occurred in the SCA group, in one patient CGH profile was not available. For the whole group at 3, 5 and 10-year OS and DFS were 0.95 (95% CI 0.81-0.99), 0.91 (95% CI 0.77-0.97) and 0.91 (95% CI 0.77-0.97), and 0.95 (95% CI 0.90-0.99), 0.92 (95% CI 0.85-0.98) and 0.86 (95% CI 0.78-0.97), respectively. DFS was significantly lower in the SCA group than in the NCA group (3-years, 5-years, and 10-years DFS 0.92 (95% CI 0.53-0.95), 0.80 (95% CI 0.40-0.95) and 0.60 (95% CI 0.16-0.87) vs 1.0, 1.0 and 1.0, respectively, p=0.005).

**Conclusions:**

The risk of treatment failure was higher in patients with SCA profile, but only in patients over 18 months. All relapses occurred in children having obtained the complete remission, with no previous radiotherapy. In patients over 18 months, SCA profile should be taken into consideration for therapy stratification as it increases the risk of relapse and this group may require more intensive treatment.

## Introduction

Neuroblastoma (NBL) is the most frequent extracranial solid tumor in children (1). It is characterized by substantial clinical heterogeneity, from spontaneous tumor regression to aggressive clinical behavior (2–4). Current risk classification utilizes clinical factors at diagnosis (age (5), disease stage (5–7) and tumor biologic features (*MYCN* status (8), DNA ploidy status (9, 10), and histopathology (11) to assign patients to appropriate therapy based on risk of treatment failure. Non-high-risk neuroblastoma (low- and intermediate-risk categories) represents nearly half of all newly diagnosed patients. Clinical experience with this system suggests that this stratification of non-high risk NBL patients for treatment may be useful, but clinical course of disease can markedly differ in patients with the same clinicopathological parameters (12, 13). Patients with stage 3 neuroblastoma according to International Neuroblastoma Staging System (INSS) represent a heterogenous group with respect to disease presentation and prognosis. All patients with *MYCN* amplification are treated according to high-risk protocols, but controversies still exist regarding the most effective treatment schedules for stage 3 patients without *MYCN* amplification. Treatment varies among study groups (14–17) and depends on age, pathology and biological features, like 1p and 11q deletions, ranging from observation (18) or surgery alone (19) to use of low or middle intensive chemotherapy (15, 16) or even treatment including high-dose therapy (HDC) with autologous stem cell rescue (ASCR) (20). In SIOPEN LINES protocol, patients with stage 3 tumors (L2 group) are treated with chemotherapy and surgery only (low risk group and intermediate risk group with favorable pathology), or surgery is followed by radiotherapy and 13-cis retinoic acid maintenance in children over 18 months with unfavorable pathology (21). The most important difference in COG strategy is inclusion of patients over 18 months with unfavorable histopathology into the high risk group and treating them according to the high risk protocols, with HDC and ASCR, radiotherapy and immunotherapy. In the new COG risk stratification system SCA profile is also incorporated as a factor of the high risk in these patients (20). Due to the presence of image defined risk factors the radical surgery is usually not possible in these patients at diagnosis (22), and sometimes also after chemotherapy. Escalation of chemotherapy, however, may lead to long-term treatment toxicities, usually not influencing possibility of tumor resection (23–26). Controversies also exist concerning the necessity of employment of radiotherapy (27). Since the outcome for some patients from this group is still not satisfactory, it is necessary to provide more precise prognostic factors to optimize treatment strategies.

The aim of the study was evaluation of genetic aberrations impact on treatment results in patients with stage 3 NBL without *MYCN* amplification and its correlation with clinical and other biological markers predictive of treatment failure.

## Patients and methods

Retrospective analysis of 40 stage 3 patients with histopathological confirmed neuroblastoma without *MYCN* amplification, diagnosed between January 1, 2000, and December 31, 2017, in 3 centers of the Polish Pediatric Solid Tumors Study Group (PPSTSG) was performed. Tumor material was available for genetic analysis from all study patients. The observation was finished in December 2020. All patients have complete follow-up data. Mean follow up was 141.3 (range 11.7-151.2) months. The prognostic value of age at diagnosis (under 18 vs over 18 months), International Neuroblastoma Pathology Classification (INPC) diagnostic category and presence of segmental or numerical chromosomes aberrations were evaluated. Biochemical markers, as neurospecific enolase (NSE), lactate dehydrogenase (LDH) activity and concentration of ferritin in serum were also analyzed, as well as catecholamines urine concentration at diagnosis.

All children received standard treatment according to current protocols, including 36 patients who received induction chemotherapy according to protocols for stage 3 NBL. During this period, three protocols for localized unresectable NBL were used, based on the SIOPEN strategy: *Treatment of children over the age of 1 year with unresectable localised neuroblastoma without MYCN amplification 2000* (16) (n=12), and *European Low and Intermediate Risk Neuroblastoma Protocol: A SIOPEN study* (LINES) (21) (n=17). In infants with localized tumors, therapy according to *European Infant Neuroblastoma Study* (4, 5, 28) was employed before the implementation of LINES protocol (n=7). One child received chemotherapy according to Japanese protocol and the other one received the therapy according to the high-risk protocol due to large tumor volume.

In SIOPEN LINES protocol, patients with stage 3 tumors (L2 group) are treated dependent on age. Patients <18 months are classified as the low risk group and their treatment depends on genomic profile (segmental chromosomal aberrations (SCA) versus numerical chromosomal aberration (NCA) and in NCA group therapy depends also on the presence of the life threatening symptoms (LTS). The therapy varies from observation (clinician’s decision, patients with NCA, no LTS) to 2 – 6 cycles of chemotherapy, followed by surgery. Patients with L2 tumors over 18 months at diagnosis are divided on the basis on pathology only and genomic profile is not included into therapy stratification. The strategy in patients with favorable histopathology is the same as for patients under 18 months, with 4 cycles of chemotherapy and surgery. In patients with unfavorable histopathology, 6 cycles of chemotherapy are given (2 cycles may be given after surgery) and surgery is followed by radiotherapy and 13-cis retinoic acid maintenance (21).

Only two children did not receive any chemotherapy and they were treated with surgery alone. In both cases, the radical tumor resection was feasible despite the presence of tumor crossing the midline (stage 3). According to International Neuroblastoma Risk Group (INRG), these tumors would be classified as L1, without the need for chemotherapy.

In 20 children, the surgery performed after initial chemotherapy was not macroscopically radical. Eleven of them had additional therapy given after the surgery – it included additional cycles of conventional chemotherapy and/or radiotherapy and/or 13-cis RA therapy. The additional off-protocol treatment did not have any influence on the size of residual tumor mass.

Radiotherapy (RTX) was used in 14 patients and in 15 patients 13-cis retinoic acid (13-cis RA) was employed, either as a part of standard protocol (LINES, group 8) or according to individual clinician’s decision. In eight children both 13-cis RA and RTX were used (in five patients as a standard therapy in LINES protocol). In four patients, spinal canal involvement was diagnosed, and two others presented with opsoclony-myoclony syndrome. Group characteristics is presented in **Table 1**, whereas the treatment modalities are presented in **Table 2**.

**Table 1:** Patients’ characteristics.

		Number of patients (n)
Age (months)		0.2 – 127.8 (mean 23.7, median 14.1)
	<18 months	25
	18-60 months	12
	≥ 60 months	3
Histopathology		
	FH	17
	UH	18
	NBL, NOS	5
Chromosomal aberrations		
	NCA	16
	SCA	12
	1p del	1
	2p gain	5
	11q del	4
	14q del	2
	17q gain	6
	No data	12
LDH		
	<2xN	31
	≥2xN	8
	Not done	1
Ferritin		
	<2xN	3
	≥2xN	19
	Not done	21
Primary tumor localization		
	Abdomen	28
	Others	12

FH, favorable pathology; UH, unfavorable pathology; NBL, neuroblastoma; NOS, non other specified; NCA, numerical chromosomal aberration; SCA, segmental chromosomal aberration; del, deletion; LDH, lactate dehydrogenase; N, norm.

**Table 2:** Treatment modalities.

Chemotherapy		38
	Localized unresectable SIOPEN	17
	LINES	12
	INFANT NB	7
	HR NBL	1
	Japanese protocol	1
Radiotherapy		14
	According to protocol	6
	Clinicians’ decision	8
13-cis retinoic acid therapy		15
	According to protocol	7
	Clinicians’ decision	8
Off-protocol therapy		15
	Chemotherapy	12
	Radiotherapy	8
	Surgery	4
	13-cis retinoic acid	8

To evaluate the genetic profile, in all children in which fresh frozen or formalin-fixed (FFPE) tumor samples were available, array comparative genomic hybridization (aCGH) for analyzing copy number variations and Sanger sequencing for *ALK* point mutations were done. It was used 200 ng of tumor DNA for aCGH analyses. The Agilent SurePrint G3 CGH ISCA v2 Microarray Kit 8x60K array platform was used for genome evaluation without enzymatic digestion. The resolution for aCGH evaluation was established on 0.15Mb for frozen samples and 1Mb for FFPE. Frozen and FFPE aCGH profiles were matched for 7 patients to confirm and validate results (29). For screening of two the most common mutations in the *ALK* gene (Phe1174Leu and Arg1275Gln) 100 ng of genome DNA was used. The sequencing was performed on the Applied Biosystems 3500 Genetic Analyzer (Thermo Fisher Scientific, Waltham, MA).

Events for the disease-free survival (DFS) analysis were defined as relapse or disease progression. Treatment failure was defined as the progression of size of the existing lesions or the occurrence of the new lesion, either distant or in the localization of primary tumor. Time to disease relapse/progression for DFS analysis was calculated as time from the date of diagnosis to the first relapse/progression or to the date of the last observation. Time to event for overall survival (OS) analysis was time from date of diagnosis until death or the date of last observation. Survival curves were estimated according to the Kaplan-Meier method (30, 31), and curves were compared using a log-rank test (p<0.05 was considered statistically significant). The analysis of the difference in markers level were done with U Mann-Whitney test and the differences between groups with chi square test.

Written informed consent for therapy was obtained from parent or legal guardians, including use of remained tumor tissue as well as clinical data for further analyses. The agreement of the Bioethical Committee of the Jagiellonian University Medical College was obtained for storage and analyses for tumor tissue (1072.6120.127.2017 and 1072.6120.239.2019).

## Results

Forty children aged 0.2-127.8 months (mean 23.7, median 14.1) were analyzed. In two cases, CGH was not performed because the samples contained a low percentage of NBL cells. In ten further samples CGH was performed, but data were not included to analyses due to not interpretable results, caused by low quality of genomic DNA extracted from FFPE. In 28 patients, a pangenomic approach was possible.

Of 40 patients, 25 (62.5%) were younger than 18 months, including 16 infants (40%), eight of them under 6 months. Only three patients were over 5 years at diagnosis. Tumor histopathology according to INPC was available in 35 out of 40 patients (87.5%), of whom 17 (48.6%; including 14 patients under 18 months) had favorable and 18 (51.4%, including five patients under 18 months) had unfavorable pathology. In children over 18 months unfavorable pathology were more common than in younger patients (p=0.001). In five other patients the tumors were described only as neuroblastoma, not other specified.

In 12 patients, segmental chromosomal aberrations (SCA) were found, including two patients under 18 months (12.7%). The most common aberration was 17q gain (six patients), followed by 2p gain (five patients) and 11q deletion (three patients). Deletion of 1p was found only in one patient. We did not observe neither 3p nor 6q loss. Numerical chromosomal aberrations (NCA) were found in 16 patients, including 14 patients at the age of below 18 months (87.5%). In children over 18 months SCA were more common (p=0.0001).

In 24 patients, both histopathological tumor classification according to INPC and CGH results were available. In seven patients over 18 months, unfavorable pathology with SCA were found (38.9%). Only three children under 18 months had SCA, including two with favorable and one with unfavorable pathology. Unfavorable pathology was significantly correlated with SCA genomic profile (p=0.04) and age over 18 months (p=0.008). Data concerning histopathological and genomic profiles are presented in **Table 3**.

**Table 3:** Histopathologic and CGH results.

Patients (n)	>18 months (n=15)	<18 months (n=25)
UH, SCA	7	1
UH, NCA	0	4
FH, SCA	1	2
FH, NCA	0	9
UH, genomic profile not available	4	2
FH, genomic profile not available	2	3
SCA, INPC not available	1	0
NCA, INPC not available	0	3
INPC and profile not available	0	1

UH, unfavorable histopathology; FH, favorable histopathology; SCA, segmental chromosomal aberrations; NCA, numerical chromosomal aberrations; INPC, International Neuroblastoma.

Results of *ALK* mutations evaluation (Phe1174Leu and Arg1275Gln) were available in 35 out of 40 patients (75%). The *ALK* mutation was found only in one patient – the infant with abdominal tumor with unfavorable pathology and SCA genomic profile with 2p gain present. He was treated with chemotherapy and surgery, and he achieved a partial response. According to the clinicians’ decision, after surgery he received additional three cycles of temozolomide/irinotecan chemotherapy, primary tumor radiotherapy and treatment with 13 -cis RA, with no complete remission of tumor. The child is alive over 5 years from diagnosis, with stable disease.

**Figure 1:**
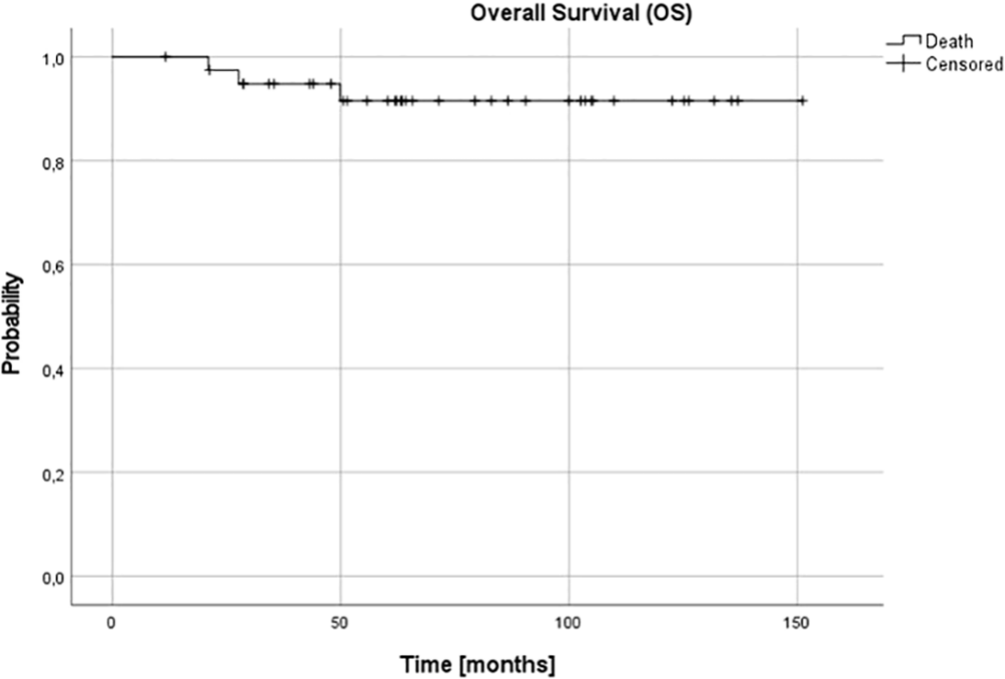
Overall survival (OS) for the whole group - patients with stage 3 neuroblastoma without MYCN amplification; 3-, 5- and 10-year OS: 0.95, 0.91 and 0.91.

**Figure 2:**
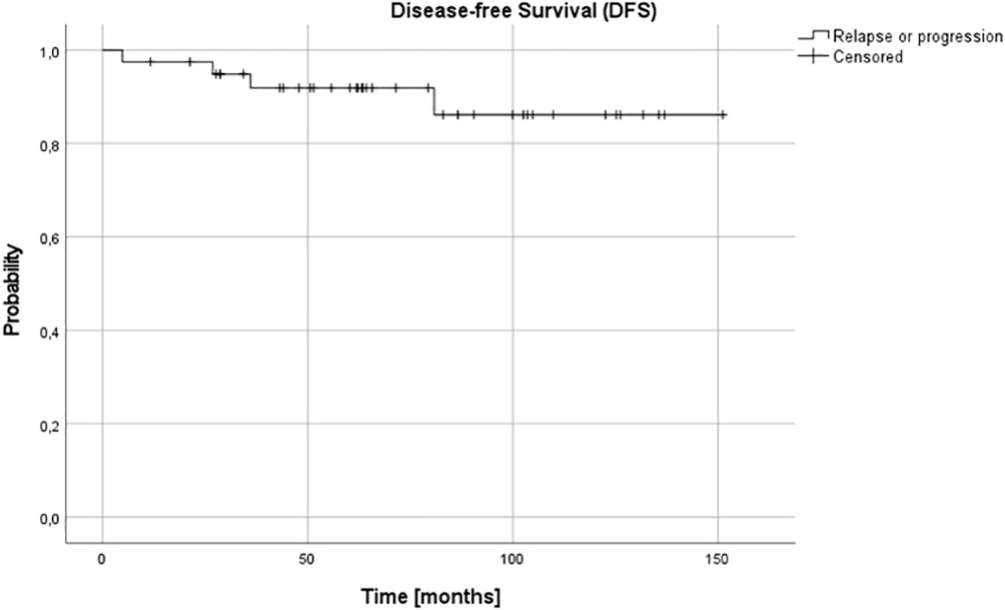
Disease-free survival (DFS) for the whole group - patients with stage 3 neuroblastoma without MYCN amplification; 3-, 5- and 10-year DFS: 0.95, 0.92 and 0.86.

For the whole group at 3 and 5, and 10 years OS and DFS were 0.95 (95% confidence interval (CI) 0.81-0.99), 0.91 (95% CI 0.77-0.97) and 0.91 (95% CI 0.77-0.97), and 0.95 (95% CI 0.90-0.99), 0.92 (95% CI 0.85-0.98) and 0.86 (95% CI 0.78-0.97), respectively (**Figures 1**, **2**).

**Figure 3:**
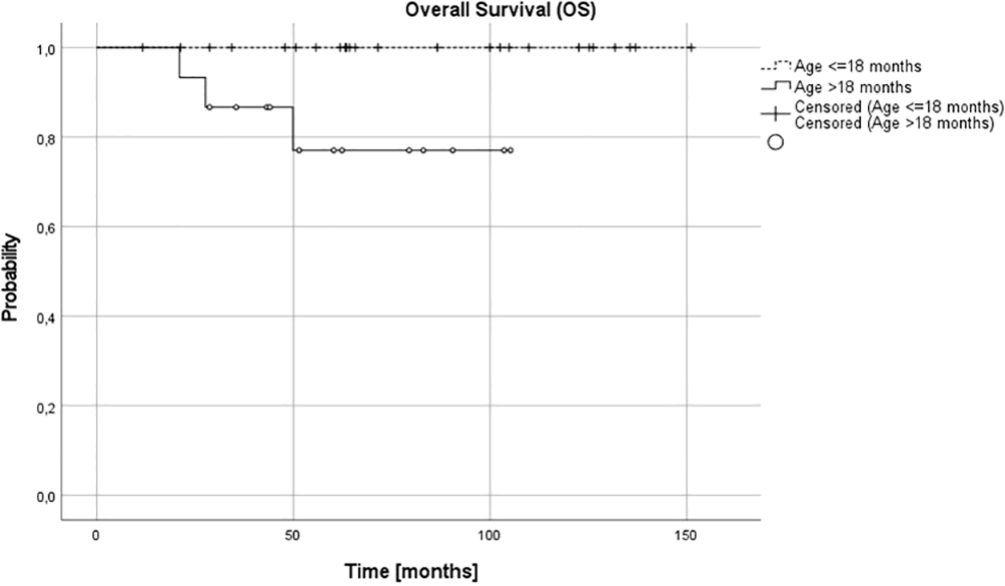
Overall survival (OS) for children over and under 18 months; 3-, 5- and 10-year OS: 0.87, 0.79, 0.79 and 1.0 in all time points, respectively (p=0.005).

**Figure 4:**
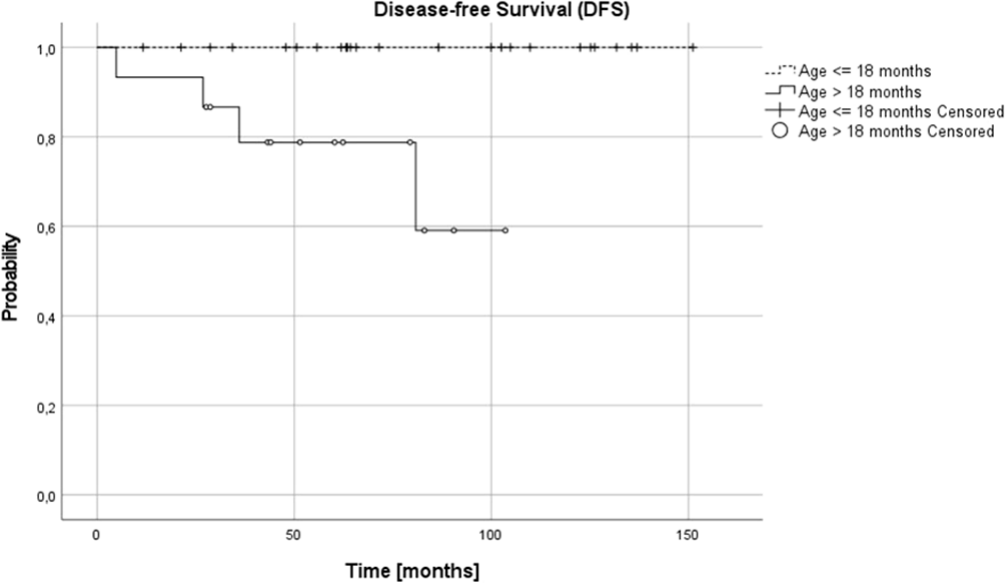
Disease-free survival (DFSS) for children over and under 18 months; 3-, 5- and 10-year DFS: 0.87, 0.79 and 0.59 and 1.0 in all time points, respectively (p=0.005).

Complete remission (CR) was obtained in 26 patients (65%), very good partial response (VGPR) in 5 patients (12.5%) and partial response (PR) in 8 patients (20%). In one (5%) patient, disease progression was diagnosed during the first line treatment. Three relapses (one distant and two local relapses) were diagnosed, all of them in children over 18 months at diagnosis. The progression occurred during therapy, five months from diagnosis, local relapses 27 and 36 months and distant relapse over 60 months from the first diagnosis. No therapy failures occurred in children with NCA profile over or under 18 months or in children under 18 months, irrespective of pathology and CGH results. Three treatment failures occurred in the SCA group, in one patient CGH profile was not available. DFS was significantly lower in the SCA group than in NCA group (3-year, 5-year, and 10-year DFS 0.92 (95% CI 0.53-0.95), 0.8 (95% CI 0.40-0.95) and 0.6 (95% CI 0.16-0.87) vs 1.0, 1.0 and 1.0, respectively, p=0.005). DFS was also significantly lower in the group with the confirmed presence of SCA in comparison to all other patients (p=0.03). The age of patients at diagnosis was the most important prognostic factor, with 3-year, 5-year, and 10-year OS 0.87 (95% CI 0.56-0.97), 0.77 (95% CI 0.43-0.92), 0.77 (95% CI 043-0.92; p=0.005) and DFS 0.87 (95% CI 0.56-0.97), 0.79 (95%CI 0.47-0.93) and 0.59 (95% CI 0.18-0.85; p=0.005) for children older than 18 months and OS and DFS 1.0 in all time points for children younger than 18 months (**Figures 3**, **4**). DFS was also lower in patients with unfavorable pathology in comparison to patients with favorable pathology (5-year DFS 1.0 in both groups, 10-year DFS 1.0 vs 0.67 (95% CI 0.05-0.95, respectively), but the difference was not statistically significant (p=0.07), probably due to the low number of events.

All relapses occurred in children having obtained the complete remission after the first line therapy. None of relapsed patients had radiotherapy in the first line treatment. In two patients relapse (one local, one disseminated) was treated according to the high risk protocol, including megachemotherapy and immunotherapy – both children are alive in the durable second remission. The third relapsed child and the child with the disease progression had the second line chemotherapy and both died during this treatment due to the further disease progression. Both had also severe toxicities caused by long-lasting chemotherapy, without significant effect of treatment on the tumor size.

Three patients died – two of them of disease progression and one of toxicity (complications of varicella-zoster infection). All deaths from disease progression occurred in the SCA group (p=0.005). The influence of pathology did not reach statistical significance, with worse prognosis in children with unfavorable pathology (p=0.09).

Biochemical parameters were evaluated, but markers analyzed at diagnosis (LDH, ferritin, NSE, catecholamines in urine) did not have statistically significant influence on OS or DFS.

In 21 children, LDH activity was lower than 2x normal activity (13 children under 18 months, 52% and eight children over 18 months, 53.3%); the difference between age groups was not statistically significant (p=0.9). The difference in numbers of patients with increased LDH activity was also not significant in dependence on INPC (p=0.2) or presence of segmental or numerical chromosomal aberrations (p=0.92). The difference between SCA/NCA groups was also not statistically significant for NSE (p=0.2), HVA (p=0,.8), VMA (p=0.55) or dopamine (p=0.5). No statistically significant differences were found also for age or pathology for any of the markers.

## Discussion

The clinical heterogeneity of NBL tumors is well known (1). Although age and tumor stage are established prognostic factors (1, 5), used alone they do not unequivocally predict the tumor behavior, especially in low and intermediate risk groups. Tumor histology (11), the *MYCN* oncogene status (8), tumor cell DNA content (9, 32) as well as genetic markers, like 11q or 1p deletions (33, 34) are well known prognostic factors. However, there is still no optimal clinical algorithm to establish the best treatment for no high-risk patients with unresectable tumors.

There are few published reports focused on the prognostic factors and the outcome exclusively in patients with stage 3 NBL without *MYCN* amplification. The survival of this group of patients is estimated for about 90%, independently on treatment modalities (14, 15, 19), but OS decreases to 82% in patients with unresectable tumor and unfavorable pathology (20). Although this survival rate seems to be satisfactory in comparison to the high-risk patients, probably there is a subgroup of patients which should benefit from more intensive therapy.

In our setting, the 5-year OS and DFS in the whole group was 91% and 92%, respectively. It was excellent in children under 18 months (5-year OS and DFS 100%), independent on other analyzed risk factors, including pathology and SCA/NCA profile, but it decreased in older patients (87% and 78%, respectively). The risk of treatment failure was higher in patients with SCA profile, but only in patients over 18 months. The number of patients with stage 3 NBL is low in different studies and these patients are usually analyzed together with other patients with localized tumors, in whom presence of SCA may have no such influence. There is high correlation between SCA and unfavorable histopathology which is better established prognostic factor, and taking into consideration low number of such patients in studies, the influence of SCA profile in these patients might have been underestimated (14–16, 19).

In most of the studies, the older age is a risk factor for poorer prognosis (35), but in other studies it does not appear to impact survival (19). The small number of patients over 18 months at diagnosis in studies and different treatment approach makes comparison between the reports difficult. In our cohort, all patients with treatment failure were over 18 months at diagnosis (four out of fifteen patients over 18 months - 26.7%). We did not observe relapses in younger patients, even with unfavorable pathology, SCA genomic profile or in one case with *ALK* mutation.

The number of young infants (under 6 months of age at diagnosis) with stage 3 tumor was also low in different studies, if reported (Modak (19) reported four young infants in the group of 53 patients.) In our cohort, we found relatively high number of children under 6 months (eight out of 40, 20%), what might influence prognosis in children under 18 months. Six out of eight young infants (75%) had favorable histopathology; five patients (62.5%) had NCA genomic profile. One infant under 6 months of age, with favorable INPC histopathology, SCA genomic profile, and *ALK* mutation remains alive with residual tumor mass over 5 years after diagnosis. *ALK* aberrations are well-known poor prognostic factor for high-risk NBL patients (36, 37), but in this very young single patient it seemed to not influence the prognosis.

Presence of SCA may be a useful prognostic marker in localized NBL, especially in unresectable stage 3 tumors, to define the group requiring more intensive therapy. The SCAs are most frequent in intermediate- and high-risk tumors due to unbalanced chromosome translocations, which are thought to arise from DNA double-strand breaks repaired erroneously, whereas abnormality in the mitotic segregation of the chromosomes is thought to exist in tumors with NCAs (38, 39). The profile of NCAs and SCAs in our cohort is concordant with the profiles described in the literature (38–40). We observed the high number of 2p gain, which was described elsewhere (41). The presence of SCA differed significantly in two patients’ groups, being significantly higher in children over 18 months, compared with younger ones. The poor prognosis was observed in older children and presence of SCA, irrespective of chromosome involved (40). It is also confirmed in our study, with treatment failures found only in patients over 18 months and SCA profile. In some protocols, like LINES, SCAs are one of the factors stratifying patients below 18 moths to more intensive therapy (21). However, in our cohort, SCAs did not influence prognosis in children under 18 months. It should be considered if they could be used as a stratification factor for the risk groups in patients over 18 months. In the revised risk classification of neuroblastoma of Children’s Oncology Group (COG), SCAs have been implemented to prospectively define treatment assignment (20).

The intensity and duration of chemotherapy prior to surgery seems to be not prognostic in the most of patients (19), and the curability of recurrent low- and intermediate-risk localized NBL is satisfactory (3). There are data confirming the limited proliferative and metastatic potential of residual post-operative disease (19) and the long-term side effects of cytotoxic therapy may be severe, especially in the youngest children (42). The presence of image-defined risk factors determines the possibility to achieve complete resection, but complete resection is not prognostic for OS (19, 43, 44). What is interesting in our cohort, all three relapses occurred in patients after complete resection of primary tumors, while leaving residual tumor mass did not seem to influence survival. What is also important, none of the patients in our group with disease relapse or progression had RTX of the primary tumor in the first line treatment. It may suggest that at least some patients could benefit from more intensive therapy, even high risk treatment as proposed by COG group (17).

There are also late relapses described in patients with primary localized NBL, over 5 years after the end of therapy (19). Also, in our cohort, we observed one distant relapse more than 5 years after primary diagnosis. Although the numbers are small, the need for continued follow-up, over the standard 5 years, should be considered especially in older children with SCA profile.

Although there are many factors identified that influence survival in stage 3 NBL without *MYCN* amplification, it is still difficult to clearly identify patients requiring more intensive therapy. In children with resectable tumors, the ability to observe patients with favorable genomic profile and favorable histology was confirmed (2) and it is still investigated (COG ANBL1232) (45). This strategy requires confirmation in patients with unresectable tumors with favorable biology. Age, histopathology, and genomic profile are the most important factors that influence the prognosis in localized NBL. In our study, for children under 18 months, neither SCA nor unfavorable pathology influenced survival.

## Conclusions

Our study shows that patients over 18 months with SCA profile have the highest risk of relapse, probably also over 5 years after the end of treatment. Prognosis is also influenced by pathology, although unfavorable pathology is significantly correlated with SCA profile. As none of relapsed patients in analyzed group had RTX in the first line treatment, it may suggest that at least some of these children could benefit from a such treatment modality. Children over 18 months with NCA profile probably do not need intensive treatment including RTX, irrespective of histopathology and the presence of residual tumor. In patients over 18 months, SCA should be taken into consideration for therapy stratification as it increases the risk of relapse and this group may require more intensive treatment.

## Data availability statement

The raw data supporting the conclusions of this article will be made available by authors according to the national data protection rules, without undue reservations. The data presented in in the study are deposited in the Array Express repository, accession number E-MTAB-12670.

## Ethics statement

The studies involving human participants were reviewed and approved by Jagiellonian University Ethical Committee.

## Author contributions

AW: original idea for the study. AW, KS, GD, and TK: data analysis and interpretation. AW and TK: statistical analyses. AW and KP-W: drafting the manuscript. AW, JS, and MU: data acquisition. AW, KS, TK, JS, MU, GD, KP-W, and WB: critically revising the manuscript for important intellectual content. All authors contributed to the article and approved the submitted version.
